# Establishment of pancreatic cancer cell lines with endoscopic ultrasound‐guided biopsy via conditionally reprogrammed cell culture

**DOI:** 10.1002/cam4.2210

**Published:** 2019-05-01

**Authors:** Hee Seung Lee, Jae Seung Lee, Jinyoung Lee, Eun Kyung Kim, Hoguen Kim, Moon Jae Chung, Jeong Youp Park, Seung Woo Park, Si Young Song, Seungmin Bang

**Affiliations:** ^1^ Division of Gastroenterology, Department of Internal Medicine Yonsei University College of Medicine Seoul Korea; ^2^ Department of Pathology Yonsei University College of Medicine Seoul Korea

**Keywords:** biopsy, cell line, drug therapy, pancreatic neoplasms, precision medicine

## Abstract

Recent studies have identified the mutational landscape of pancreatic cancer and suggested tumor‐specific subtypes. However, the major hurdle against personalized treatment is the difficulty to obtain sufficient cancer tissues from most inoperable cases. We investigated whether patient‐derived conditionally reprogrammed cells (CRCs) can be constructed using a small piece of tumor tissue using endoscopic ultrasound (EUS)‐guided fine needle biopsy (FNB). Thirty patients with pancreatic solid mass (mean size, 34.6 mm) were enrolled prospectively. Among 22 patients who were diagnosed with pancreatic ductal adenocarcinoma, we established patient‐derived pancreatic cancer cell lines from eight patients (36.4%). Immunofluorescence colony staining for CRCs showed that the cytoplasm of cancer cells was clearly stained with anti‐cytokeratin 19 monoclonal antibody. In the soft agar colony formation assay, CRCs formed colonies compared with the negative control by day 15. In vivo, implanted CRCs showed tumor engraftment and hematoxylin and eosin staining showed pancreatic cancer ductal structure. All established CRCs showed a KRAS mutation. In conclusion, we established patient‐derived pancreatic cancer cell lines with a small tumor tissue obtained by EUS‐FNB. With in vitro drug sensitivity and genomic studies, established patient‐derived cell lines can be used in identification of new targets for diagnosis and treatment of pancreatic cancer.

## 1 INTRODUCTION

Recent advances in cancer genomics have driven a paradigm change in anticancer treatment toward personalized therapy according to the genetic signature of each patient.^1^, ^2^ Next generation sequencing methods have identified genetic changes, including single nucleotide variants, and have allowed the classification of subtypes of pancreatic cancer according to their different treatment responses. To utilize these genetic features of pancreatic cancer, various recent preclinical models were introduced to pancreatic cancer research.^3^ The successful application of preclinical cancer models derived from tumor tissue provides the opportunity for personalized drug selection and prognosis prediction.

For the establishment of preclinical cancer models, sufficient tumor tissue acquisition is essential. However, obtaining surgical specimens is extremely difficult in pancreatic cancer because only less than 15% of all patients with pancreatic cancer can be treated using surgical resection. The only reliable way to obtain the tissue sample is endoscopic ultrasound (EUS)‐guided fine needle biopsy (FNB) in most patients with advanced pancreatic cancer.^4^ However, the amount of tissue obtained using EUS‐FNB is not enough to perform advanced laboratory or molecular analysis, including exome sequencing. Therefore, the establishment of a platform of primary cancer cell lines with the small piece of tumor tissue obtained using EUS‐FNB is urgently required.

In this study, we aimed to investigate whether patient‐derived preclinical cancer models can be constructed using a small piece of tumor tissue obtained via EUS‐FNB in pancreatic adenocarcinoma.

## 2 MATERIALS AND METHODS

### 2.1 Patients

Patients who underwent EUS‐guided tissue acquisition from a solid pancreatic mass at Severance hospital, South, Korea were prospectively enrolled from July 2016 to November 2017. All pancreatic masses were previously diagnosed using computed tomography (CT), magnetic resonance imaging, or previously performed EUS. The inclusion criteria were as follows: (a) patients with a solid pancreatic mass that was visualized with EUS and was above 1 cm in size in imaging studies, (b) at least 20 years of age, and (c) provided written informed consent. This study was performed in accordance with the ethical guidelines of the 1975 Declaration of Helsinki and was approved by the institutional review board of the ethics committee of Severance Hospital and registered in a clinical trial database (ClinicalTrials.gov identifier: NCT03017599). All experiments were performed in accordance with relevant guidelines.

### 2.2 Procedural technique

EUS‐FNB was performed using a convex array endosonoscope (GF‐UCT260; Olympus Co., Tokyo, Japan). Tissue acquisition was performed using a 20‐gauge needle (EchoTip^®^ ProCore^TM^ Endoscopic Ultrasound Needle, Cook Medical Inc, Bloomington, IN). Procedures were performed by five experienced endoscopists who had at least 5 years of experience and had each previously performed >500 endoscopic retrograde cholangiopancreatography procedures. Once the target lesion had been penetrated by the needle, the stylet was removed and negative suction pressure was applied for 10‐20 seconds using a 10 mL syringe while more than 20 to‐and‐fro movements within the lesion were made. Suction was then released by closing the lock of the syringe, and the needle was finally removed. After individual passes, the aspirated specimen was expelled onto a glass slide by reinsertion of the stylet. The specimen was fixed in formalin after being immersed in normal saline to decrease the amount of blood if sufficient specimen size was confirmed by the endoscopist.

### 2.3 Pathologic assessment of the obtained sample

One experienced pathologist, blinded to the clinical information, made the pathologic diagnosis and evaluation of the specimen quality for this study. A histologic core was defined as an architecturally intact piece of tissue measuring at least 550 µm in its greatest axis (corresponding approximately to the diameter of a high power microscopic field).^5^, ^6^ The pathology reports were divided into the following categories: (a) inadequate for diagnosis; (b) benign or atypical epithelium; (c) pancreatic duct adenocarcinoma or other neoplasm (a, b; negative for malignancy and c; positive for malignancy).

The quality of obtained samples was evaluated using hematoxylin and eosin staining and the following three items were examined: quantity of tissue, degree of contamination, and amount of blood in the specimens. The quantity of tissue was assessed using the scoring system described by Gerke et al^7^ Briefly, a score of zero was a sample with no material, scores of 1‐2 were samples that enabled cytological evaluation but did not provide histologic information, and scores of 3‐5 were samples that enabled histologic assessment.^8^, ^9^ A final diagnosis of the pancreatic mass was made based on the histologic diagnosis in patients with surgical or biopsy specimens or on clinical follow‐up for at least 6 months in patients with undetermined histologic results.

### 2.4 Conditionally reprogrammed cell method

To generate patient‐derived pancreatic cell lines, we used the CRC method developed by the Schlegel group at Georgetown. All tumor tissues obtained from the endoscopy room were placed in a medium containing antibiotics. Using a set of forceps and a scalpel, residual fat tissue was removed. Tumor tissues were minced into 1‐2 mm small fragments with sterile scissors. Primary cell line isolation was initiated within 1‐2 hours of tumor resection. Tissue samples were cocultured with J2 murine fibroblast feeder cells and medium containing the Rho‐kinase inhibitor (Y‐27632), as previously described.^10^, ^11^

### 2.5 Immunofluorescence assay

Cells were fixed in 4% paraformaldehyde for 15 minutes at room temperature and washed in PBS 3 times, followed by blocking with 5% normal goat serum for 1 hour and incubation with primary antibodies at room temperature (1 hour), washing three times in PBS, and incubation with second antibody for 30 minutes. The following primary antibodies were used for immunostaining: a‐amylase (A8273, rabbit polyclonal, dilution 1:100; Sigma, St. Louis, MO), cytokeratin 19 (CK19) (A53‐B/A2: sc‐6278, mouse monoclonal, dilution 1:100; Santa Cruz Biotechnology, Santa Cruz, CA), insulin (mouse monoclonal, dilution 1:100; Zymed, Waltham, MA), and vimentin (V9: sc‐6260, mouse monoclonal, dilution 1:100; Santa Cruz Biotechnology). The cells were stained with Alexa Flour conjugated secondary antibodies from Invitrogen.

### 2.6 In vitro and in vivo tumorigenicity

To evaluate tumorigenicity, in vitro tumorigenesis was examined using soft agar culture. The anchorage‐independent growth of CRCs was evaluated using a colony formation assay in soft agar. Soft agar colony formation assays were performed using the double‐layer soft agar method. In each well of a 6‐well plate, 5 × 10^4^ cells were plated on the top agar (0.5% agarose gel) over a base agar (1% agarose gel). After 2‐3 weeks of incubation in soft agar, the average numbers of colonies formed by CRCs were checked.

To evaluate in vivo tumorigenicity, 2 × 10^6^ CRCs in 0.2 mL of Matrigel (BD Biosciences) were injected subcutaneously into the flank regions of 5‐week‐old male nonobese diabetic/severe combined immunodeficiency (NOD/SCID) mice (Charles River Laboratories, Tokyo, Japan). Animals were housed at the Yonsei University animal care facility according to institutional guidelines. Monitoring for tumor growth was performed up to 3 months postimplantation. Tumor size was assessed by measurement with calipers once per week. Tumors were harvested once they reached 1.5 cm in diameter.

### 2.7 KRAS mutation analysis by polymerase chain reaction

*KRAS* mutations, key mutations in pancreatic cancer, were analyzed using polymerase chain reaction (PCR). Genetic analysis of the *KRAS* gene was performed using PCR amplification of exon 1 (codons 12 and 13), followed by direct sequencing of the PCR products. DNA was extracted using QIAGEN QIAamp^®^ DNA Mini Kits (Hilden, Germany). PCR primers for *KRAS* sequencing were as follows: forward, 5'‐aggcctg ctgaaaatga ctga‐3'; and reverse, 5'‐ggtcctgcac cagtaatatg ca ‐3' (length: 164 bp). Each PCR mix contained forward and reverse primers (each 10 pmol), 200 µmol/L of of dNTP, 1 × PCR buffer (10 mmol/L tris‐HCl (pH 8.3), 50 mmol/L KCl, 1.5 mmol/L MgCl_2_, 1 U of r‐Taq DNA polymerase (TaKaRa Taq^TM^, Takara Bio Inc, Japan), and 1 μL of DNA in a total volume of 25 μL. PCR conditions consisted of initial denaturation at 95°C for 5 minutes; 30 cycles of 95°C for 30 seconds, 60°C for 30 seconds, and 72°C for 30 seconds; and final extension at 72°C for 7 minutes. General sequencing was performed at COSMO Genetech (Cosmo Genetech Co., Ltd., Korea), and analyzed using an ABI 3730 (Applied Biosystems, USA).

### 2.8 Statistical analysis

Continuous variables were expressed as mean ± SD, and categorical variables were expressed as proportions, n (%). A *P* < 0.05 on a Mann‐Whitney U test indicated statistical significance. Statistical analyses were performed using spss version 20.0 (SPSS, Chicago, IL).

## 3 RESULTS

### 3.1 Patient characteristics

**Figure 1:**
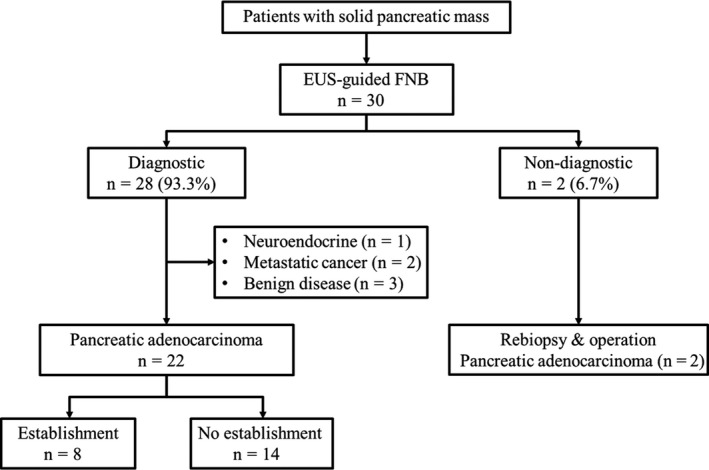
Flow chart. Diagnostic was defined as obtaining specimens sufficient for histologic interpretation. Nondiagnostic was defined as obtaining tissues insufficient for histologic interpretation. EUS, Endoscopic ultrasound; FNB, fine needle biopsy

**Figure 2:**
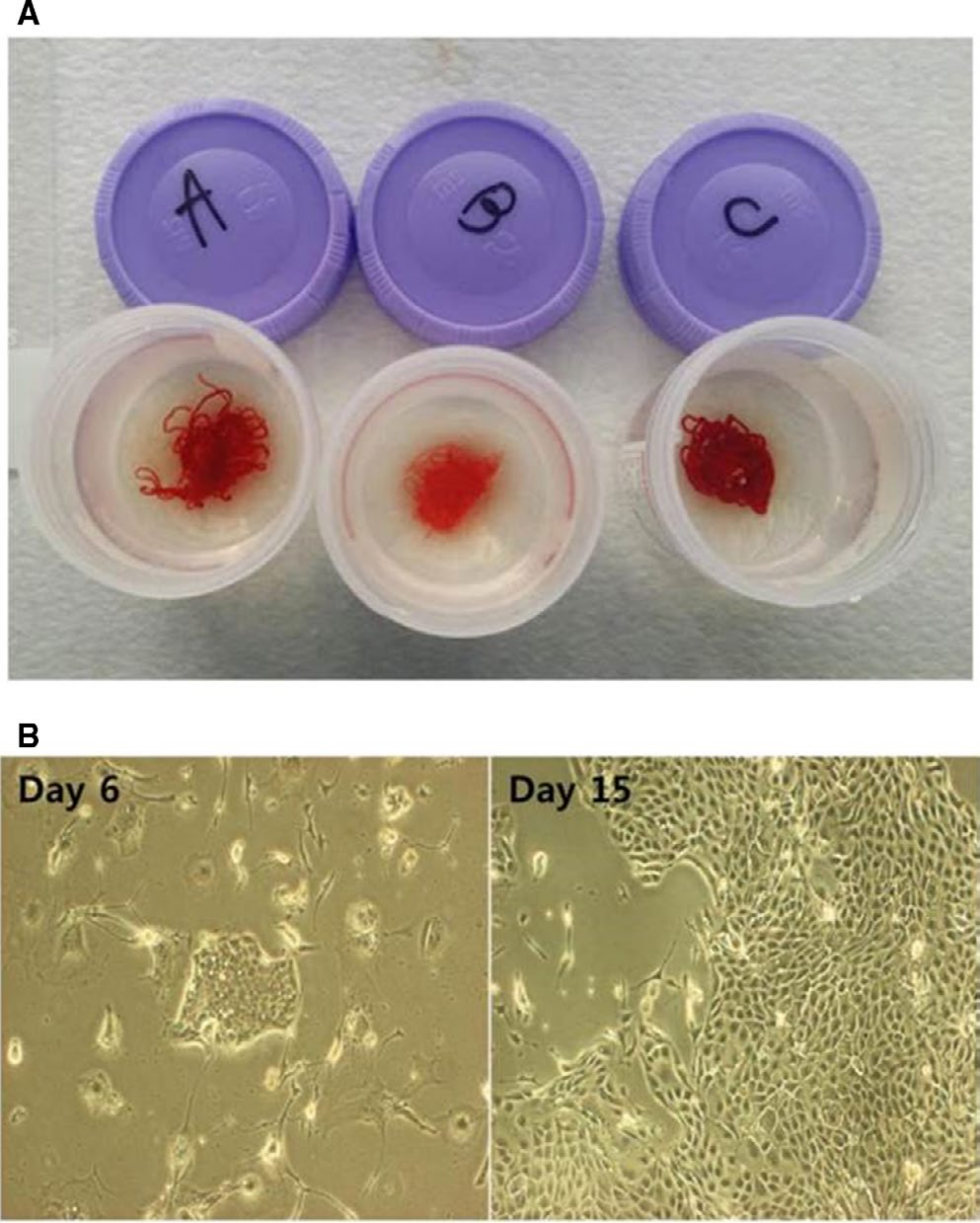
Representative specimens. (A), All specimens were grossly verified to ensure that they were suitable for obtaining a histology‐based diagnosis by the endoscopist. (B), Conditionally reprogrammed cell formation after 15 days of culture

A total of 30 patients who underwent EUS‐FNB were prospectively enrolled in this study (Figure 1). The characteristics of the patients and pancreatic solid masses are summarized in Table 1. Their mean age was 65.2 years (men, 53.3%). Lesions were located in the pancreatic head or neck (14 cases, 46.7%), body (10 cases, 33.3%), and tail (six cases, 20%). The mean size of the pancreatic masses was 34.6 ± 12.3 mm (range 14.2‐69.0 mm). Among the 30 enrolled patients, 24 (80%) were finally diagnosed with pancreatic ductal adenocarcinoma (PDAC). The technical characteristics and outcomes of EUS‐FNB are summarized in Table 2. The procedure was feasible without any technical failure, and all specimens were grossly adequate tissues to obtain a histology‐based diagnosis (Figure 2A).

**Table 1:** Patient demographic and pancreatic mass characteristics

Variables	Patients (n = 30)
Age, mean ± SD, (y)	65.2 ± 9.2
Men, no. (%)	16 (53.3%)
Location of mass, no. (%)	
Head or neck	14 (46.7%)
Body or tail	16 (53.3%)
Size of mass on EUS, (mm)	
Mean ± SD	34.6 (12.3)
Median	32
<20 mm, n	2 (6.7%)
≥20 mm, n	28 (93.3%)
Final diagnosis, no. (%)	
Malignancy	
Pancreatic ductal adenocarcinoma^a^	24 (80.0%)
Neuroendocrine carcinoma	1 (3.3%)
Metastatic cancer^b^	2 (6.7%)
Benign disease	
IgG4‐related disease	1 (3.3%)
Autoimmune pancreatitis	1 (3.3%)
Chronic pancreatitis	1 (3.3%)

Abbreviation: EUS, Endoscopic ultrasound.

a Two patients were diagnosed with pancreatic adenocarcinoma in the second study‐free EUS‐FNB and open pancreatic biopsy.

b Metastatic cancer originated from lung cancer and Klatskin tumor.

**Table 2:** Technical characteristics and outcomes of EUS‐guided FNB

Variables	Patients (n = 30)
Access route, no. (%)	
Transgastric	13 (33.3%)
Transduodenal	17 (56.7%)
No. of needle passes, mean ± SD	2.5 ± 0.5
2 times, n	16 (53.3%)
3 times, n	14 (46.7%)
Failure to achieve diagnosis	2 (6.7%)
Diagnostic failure	2 (6.7%)
Technical failure	0 (0.0%)
Diagnostic accuracy	28 (93.3%)
Differentiation confirmed	15 (50.0%)
Well differentiated	1 (3.3%)
Moderately differentiated	12 (40.0%)
Poorly differentiated	2 (6.7%)
Complications	3 (10.0%)
Pancreatitis	2 (6.7%)
Fever	1 (3.3%)

Abbreviations: EUS, Endoscopic ultrasound; FNB, Fine needle biopsy; No, Number; SD, Standard deviation.

### 3.2 Acquisition of the qualified and sufficient tissue

The quality of the obtained specimens is summarized in Table 3. Sufficient quantity of tissues for histologic interpretation (determined as the tissue quantity score from 3 to 5) was shown in 93.3% of cases (28/30). In a patient with a tissue quantity score of 2, the cytological interpretation was suspicious adenocarcinoma, and he was histologically diagnosed with moderately differentiated adenocarcinoma in the second EUS‐FNB. The rate of the degree of contamination lower than 25.0% was 86.7% (26/30), and the amount of blood in the specimen was minimal in 30.0% (9/30) and moderate in 36.7% (11/30) of cases.

**Table 3:** Quality of histologic specimen (n = 30)

*Quantity of tissue*	
0: sample with no material, n	0
Cytology (1‐2), n (%)	2 (6.7%)
1: sufficient material for limited cytologic interpretation but probably not representative, n	1
2: sufficient material for adequate cytologic interpretation but insufficient for histologic information, n	1
Histology (3‐5), n (%)	28 (93.3%)
3: sufficient material for limited histologic interpretation, n	8
4: sufficient material for adequate histologic interpretation but a low quality sample, n^a^	9
5: sufficient material for adequate histologic interpretation and a high quality sample, n^b^	11
*Contamination*	
1: contamination present in >50% of the slide, n	1 (3.3%)
2: contamination present in 25‐50% of the slide, n	3 (10.0%)
3: contamination present in <25% of the slide, n	26 (86.7%)
*Amount of blood*	
1: significant, n	10 (33.3%)
2: moderate, n	11 (36.7%)
3: minimal, n	9 (30.0%)

a Low quality sample, total material within a 10 power field in length.

b High quality sample, total material more than a 10 power field in length.

EUS‐FNB showed a diagnostic accuracy of 93.3% (28 of 30 cases); this consisted of 25 cases of pathologically confirmed malignancy (PDAC, 22 cases) and three cases of benign diseases (autoimmune pancreatitis, chronic pancreatitis, and IgG4‐related disease). Diagnostic failure occurred in two patients. One of these patients could not be diagnosed pathologically, even in the second study‐free EUS‐FNB trial. However, the patient was diagnosed with PDAC using direct biopsy under laparotomy in operating room and received chemotherapy. Another patient was diagnosed with a few strips of atypical epithelium based on a pathological result; however, this patient was diagnosed with PDAC in the second study‐free EUS‐FNB and also received chemotherapy (Table 2).

### 3.3 Establishment of patient‐derived pancreatic cancer cell lines

Among 22 patients who were diagnosed with PDAC via EUS‐FNB, we established patient‐derived pancreatic cancer cell lines in eight patients (Figure 1). Table 4 shows the characteristics of established CRCs using EUS‐FNB. Epithelial colonies were readily observed at 2 days and rapidly proliferated to reach confluence in approximately 7‐14 days (Figure 2B). The karyotypes of patient‐derived CRCs were highly aneuploid, and it was maintained even when passing the passage (Figure S1). It demonstrates the genomic stability in CRCs methods. Whole exome sequencing kept representative key mutations including KRAS, p53, and CDKN2A (H.S. Lee, S.J. Park, J. Lee, M.J. Chung, J.Y. Park, S.W. Park, S.Y. Song, J.M. Han, S. Bang, unpublished data).

**Table 4:** Clinical and pathological characteristics of the established 8 patients with pancreatic ductal adenocarcinoma

Number	Sex	Age	Location	Tumor size, mm	Pathology	Established cancer cell lines	Cell surface marker IF staining				In vitro tumorigenesis	In vivo tumorigenesis	KRAS PCR
							anti‐CK19	anti‐Vimentin	anti‐Amylase	anti‐Insulin			
1	Female	61	Body	33	PDAC	Yes	O	x	x	x	Yes	Not done	missense mutation, p.G12V
2	Male	53	Head	50	PDAC	Yes	O	x	x	x	Yes	Yes	missense mutation, p.G12D
3	Female	80	Head	31	PDAC	Yes	O	x	x	x	Yes	Not done	missense mutation, p.G12D
4	Male	57	Head	20	PDAC	Yes	O	x	x	x	Yes	Not done	missense mutation, p.G12D
5	Female	71	Tail	37	PDAC	Yes	O	x	x	x	Not done	Not done	missense mutation, p.G12V
6	Male	63	Body	32	PDAC	Yes	O	x	x	x	Yes	Not done	missense mutation, p.G12D
7	Male	59	Body	60	PDAC	Yes	O	x	x	x	Yes	Not done	missense mutation, p.G12R
8	Male	62	Body	58	PDAC	Yes	O	x	x	x	Yes	Not done	missense mutation, p.G12D

Abbreviations: IF, immunofluorescence; PCR, polymerase chain reaction; PDAC, pancreatic ductal adenocarcinoma.

**Figure 3:**
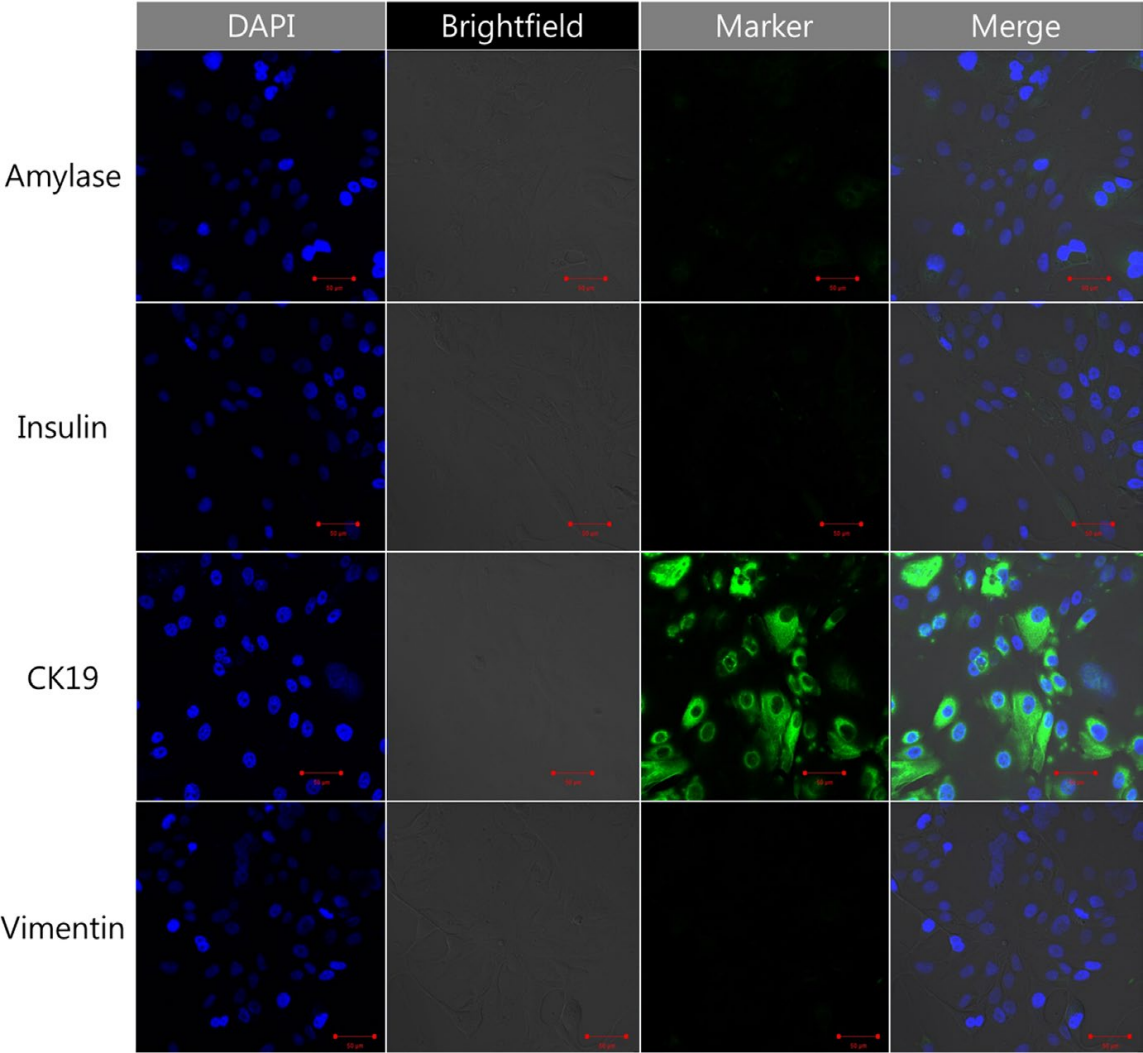
Characterization of the intensely fluorescent cells in conditionally reprogrammed cells. α‐amylase (sc‐25562, rabbit polyclonal; Santa Cruz Biotechnology), dilution 1:100, resolution rate 200x; cytokeratin 19 (A53‐B/A2: sc‐6278, mouse monoclonal; Santa Cruz Biotechnology), dilution 1:100, resolution rate 200x; insulin (sc‐8033, mouse monoclonal; Santa Cruz Biotechnology), dilution 1:100, resolution rate 200x; vimentin (V9: sc‐6260, mouse monoclonal; Santa Cruz Biotechnology), dilution 1:100, resolution rate 200x

We characterized and confirmed the CRCs by the intense fluorescence of CK19 and tumorigenesis in vitro and in vivo. Immunofluorescence colony staining for CRCs was performed using an anti‐CK19 monoclonal antibody. The cytoplasm of cancer cells was clearly stained with this antibody. However, CRCs were not stained with amylase and insulin (Figure 3).

**Figure 4:**
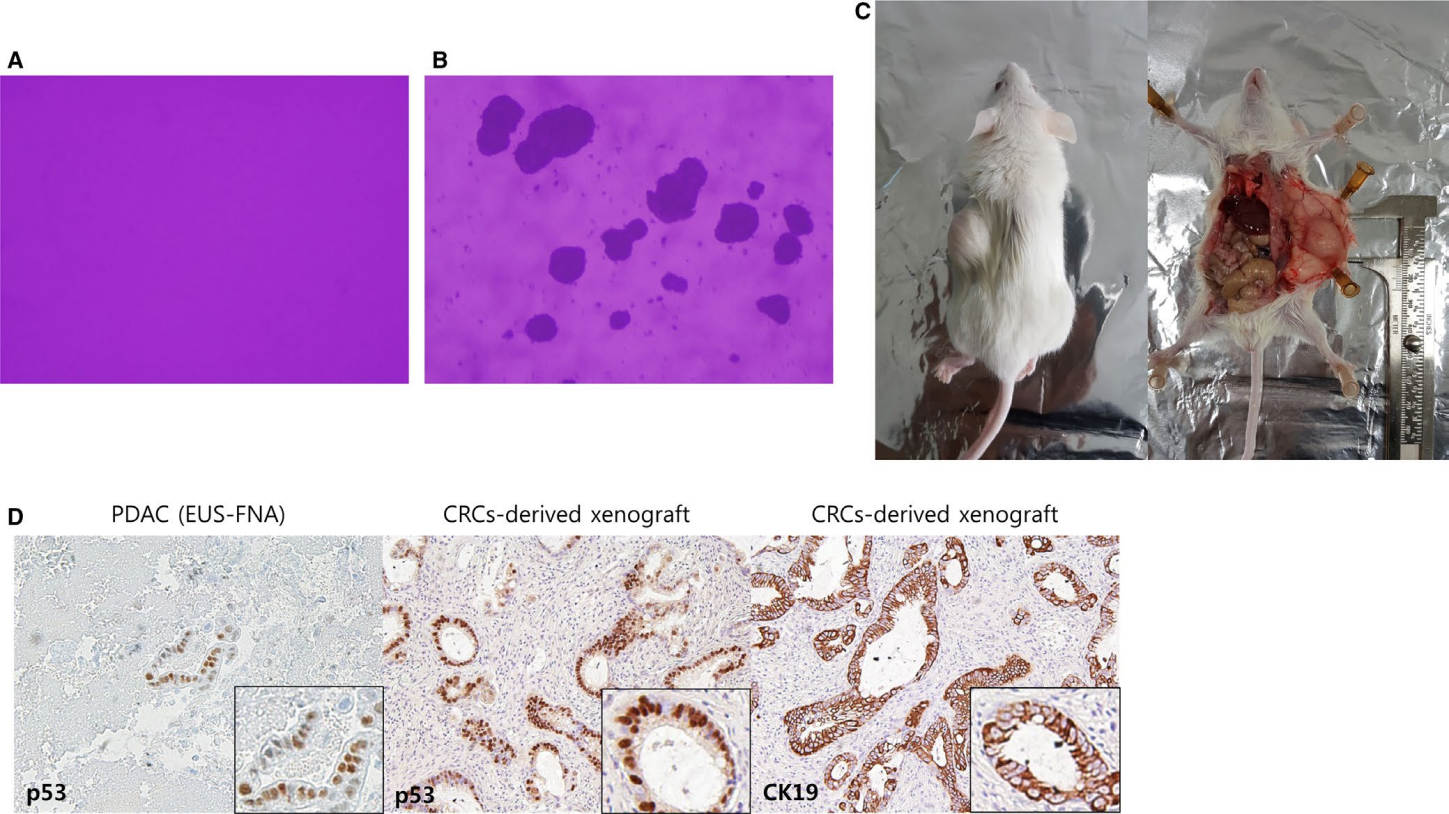
Tumorigenesis in vitro and in vivo. (A, B), In vitro tumorigenesis, Soft agar colony formation assay. (A) Negative control, (B) tumor cell formation at day 15; (C‐E) In vivo tumorigenesis and representative histology of xenografts. Hematoxylin and eosin staining showed that the implanted patient‐derived CRCs had a ductal structure. (C) In vivo tumorigenesis, 5‐week‐old male nonobese diabetic/severe combined immunodeficiency mice; (D) CRC‐derived xenograft tissue demonstrated homology for p53 expression compared with matched primary biopsy cancer tissue. The Ductal epithelial marker CK19 is positive in CRC‐derived xenograft by IHC stain

In the soft agar colony formation assay, CRCs formed colonies compared with the negative control by day 15 (Figure 4A,B). In vivo, CRCs were implanted into the NOD/SCID mice. Implanted CRCs showed successful tumor engraftment and a 15 mm sized transplanted tumor was palpated on the side of the mouse after three months. Grafted tumors were surgically removed. CRC‐derived xenograft tissue demonstrated homology for p53 expression compared with matched primary biopsy cancer tissue. And, sample had strong staining of ductal epithelial marker CK19, confirming that xenograft sample was origined from huam ductal adenocarcinoma. (Figure 4C‐D).

We cultured CRCs over 20 passages after establishment. Initial experiments related to establishment were performed within two passages including immunofluorescence. Mouse implantation was performed at 15 passages.

Regarding key oncogenic mutations, *KRAS* mutation analysis was performed using PCR. All eight CRCs established using EUS‐FNB showed a codon‐12 *KRAS* mutation: G12D (n = 5), G12V (n = 2), G12R (n = 1) (Table 4). Research for patient‐derived model establishment and drug sensitivity testing using established conditionally reprogrammed cell lines is currently ongoing and holds promising preliminary results (H.S. Lee, S.J. Park, J. Lee, M.J. Chung, J.Y. Park, S.W. Park, S.Y. Song, J.M. Han, S. Bang, unpublished data). We performed targeted deep sequencing to confirm the identity of the genetic feature of CRCs compared to the original tumor. In the data, KRAS mutation signature of the CRCs (p.G12D) correlated with the KRAS mutation found in the patient's primary tumor (p.G12D).

## 4 DISCUSSION

Most pancreatic cancer patients are ineligible for the only curative treatment, surgical resection. Therefore, it is important to establish pancreatic cancer cell lines using pancreatic biopsy samples for further molecular analysis and finding potential therapeutic targets. In this study, pancreatic cancer conditionally reprogrammed cell lines were successfully and rapidly created by means of EUS‐FNB sampling.

We checked the *KRAS* mutation in CRCs to confirm whether the CRCs reflect the original characteristics of PDAC. In previous studies, activated pathogenic variants in the proto‐oncogene *KRAS* were present in 90% of patients with PDAC,^1^, ^2^ and the identified *KRAS* mutation was representative in PDAC with diagnostic and prognostic significance. Several studies have validated the preclinical cell line models by identifying the key mutation, *KRAS.* 12, 13, 14 In this study, an activated *KRAS* mutation was present in 100% of all established CRCs. Even if the results were not described in this manuscript, we performed whole exome sequencing for five patients (Hee Seung Lee, unpublished data). We confirmed that four patients showed additional p53 mutation besides KRAS and one patient had p53 and CDKN2A mutation.

**Figure 5:**
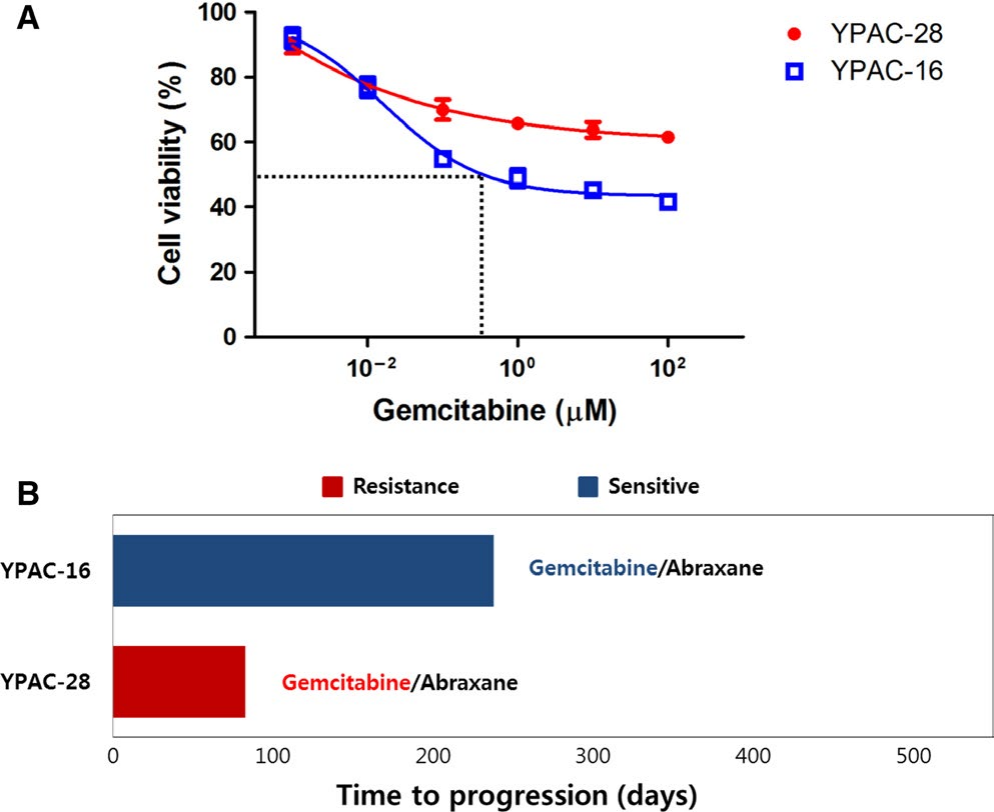
Patient‐derived conditionally reprogrammed cell lines reveal heterogeneity of chemotherapy response. (A), The growth of CRCs was analyzed via an MTT assay after treatment with various concentrations of gemcitabine over a time‐course (0‐72 hours). YPAC‐16 was more sensitive to the growth‐inhibitory effect of gemcitabine (IC50 <1 μmol/L) than YPAC‐28 (IC50 >100 μmol/L). MTT assay, 3‐(4,5‐dimethylthiazol‐2yl)‐2,5‐diphenyltetrazolium bromide (B) YPAC‐16 which was sensitive to gemcitabine showed better progression free survival than YPAC‐28 (PFS, 238 vs 83 days)

Because of intertumor heterogeneity, different patient‐derived models had dissimilar genomic properties and treatment response even if most cell lines shared the key oncogenic KRAS mutation with their original tissue. Patient‐derived cell lines showed different drug responses to the therapeutic agent. To determine the inhibitory effects of gemcitabine on each CRC’s proliferation, we measured the IC50 of gemcitabine. YPAC‐16 was more sensitive to the growth‐inhibitory effect of gemcitabine (IC50 <1 μmol/L) than YPAC‐28 (IC50 >100 μmol/L) (Figure 5). After all, progression free survival of patients who underwent gemcitabine‐based palliative chemotherapy was comparable to the results of drug screening assay (Figure 5). Therefore, patient‐derived cell lines establishment may be a fundamental tool for personalized diagnosis and treatment in PDAC. This FNB sampling‐derived CRC creation is of great importance because we will ultimately reach personalized treatment via drug screening or toxicity test.

Until now, several preclinical models have been used for drug sensitivity tests, subtype specific functional analysis, and drug development for various cancers, including PDAC.^3^, ^15^, ^16^ Previous studies have reported their results using patient‐derived xenografts (PDX) as representative preclinical models. However, PDX takes many months to establish and is difficult to use for high‐throughput screening in cancer patients.^3^ Because of these limitations, a recent patient‐derived cancer cell line model, CRCs, are extensively used in cancer research.^11^, ^15^, ^17^, ^18^ CRCs induce the propagation and immortalization of human tumor epithelial cells and generate patient‐specific cell lines within 2 or 4 weeks.^10^, ^11^ Considering that small tissues from biopsy can be a source of primary cell lines using the CRC technique, EUS‐FNB should also be evaluated as a source of primary cancer cell lines.

We need to know which EUS‐related component is associated with the improved performance of EUS‐guided tissue acquisition for establishment of primary cancer cell lines. With regards to optimal needle type, this study showed that a 20‐gauge needle was suitable for obtaining histologic core via EUS and developing the patient‐derived cell line model. In a previous study, to increase the diagnostic accuracy and acquire a histologic core, a side port needle was introduced.^19^ Further, a newly designed needle featuring the ProCore^TM^ reverse bevel technology showed an additive effect for adequate tissue acquisition.^20^, ^21^ Even though several studies reported the appropriate EUS‐guided biopsy needle to obtain a sufficient tumor sample in pancreatic cancer, those studies did not show the detailed quality of the samples using an objective score.^4^ In the present study, we showed relatively high diagnostic accuracy (93.3%) using the 20‐gauge needle, and a more favorable result with regards to obtaining the histologic core (93.3%). One of the reasons for the successful histologic core acquisition is the structural feature of the 20‐gauge needle with its side hole and cutting bevel. Further, the improved flexibility of the needle helped endoscopists to handle it. With regards to the optimal number of needle passes, the relationship between the quality of tissue and the total number of passes is shown in Table S1. There was no statistically significant difference associated with the acquisition of the histologic core according to the number of needle passes after two times (two times vs three times, *P* = 0.580). In the future, well‐designed prospective studies are needed to confirm the independent factors related to CRCs formation.

Most preclinical pancreatic cancer models were established using the tissue samples from surgical resection. With recent technological improvements in EUS‐guided biopsy, several studies have tried to use biopsy samples in pancreatic cancer research. Gleeson et al^22^, ^23^, ^24^ showed that EUS‐guided tissue sampling revealed a majority of key oncogenic mutations in pancreatic cancer and the result was paired with surgical resection specimens through targeted next generation sequencing.^22^ Allaway et al^23^ developed PDX models using EUS‐guided biopsy in 29 patients who were diagnosed with PDAC and performed genomic characterization for drug screening. Using in vitro drug sensitivity and genomic studies, establishing patient‐derived cell lines via EUS‐FNB may help to identify and validate new targets or markers for the diagnosis and treatment of PDAC in the future.

To our knowledge, the present study is the first prospective study to use EUS‐guided biopsy samples via CRC technique to establish a patient‐derived cancer model in pancreatic adenocarcinoma. A limitation of the study is that despite washing the specimens with saline to decrease the amount of blood in the specimen, the proportion of patients with significant amount of blood in the specimen was 33.3%, although it did not prevent pathologic interpretation and the establishment of patient‐derived cell lines. The to‐and‐fro movement of the FNB needle for >20 times may explain the amount of blood in the specimen. Second, the present study has a small sample size. However, this study is a pilot study to establish patient‐derived pancreatic cancer cell lines from small biopsy samples using a CRCs technique. Large sample size studies using CRCs technology are currently undergoing with whole genome analysis for patient‐derived CRCs. Finally, less number of samples (<3 samples) were used for in vivo tumorigenesis and chemosensitivity test in this study. We studied this examination of patient‐derived cell lines establishment by EUS‐FNB and confirmation of 20G needle sufficiency for cell line formation. Only representative cases were used for typical findings and confirmation. Using more surgical tissue, metastatic liver tissue, and EUS‐biopsy, CRC establishment will warrant the successful use of patient‐derived subclinical model in the future.

In conclusion, EUS‐FNB is a reliable and accurate diagnostic test for malignant pancreatic lesions, especially for the establishment of preclinical models in patients with PDAC. It may be utilized for anticancer drug screening and genomic studies for personalized treatment. With in vitro drug sensitivity and genomic studies, established patient‐derived cell lines can be used in the identification of new targets for diagnosis and treatment of pancreatic cancer.

## DISCLOSURE STATEMENT

The authors declare that they have no competing interests.
