# Supplementary figures and images for: Establishment of pancreatic cancer cell lines with endoscopic ultrasound‐guided biopsy via conditionally reprogrammed cell culture
Source: Cancer Med. 2019 May 1;8(7):3339–48. doi: 10.1002/cam4.2210 (PMC6601705; doi:10.1002/cam4.2210)

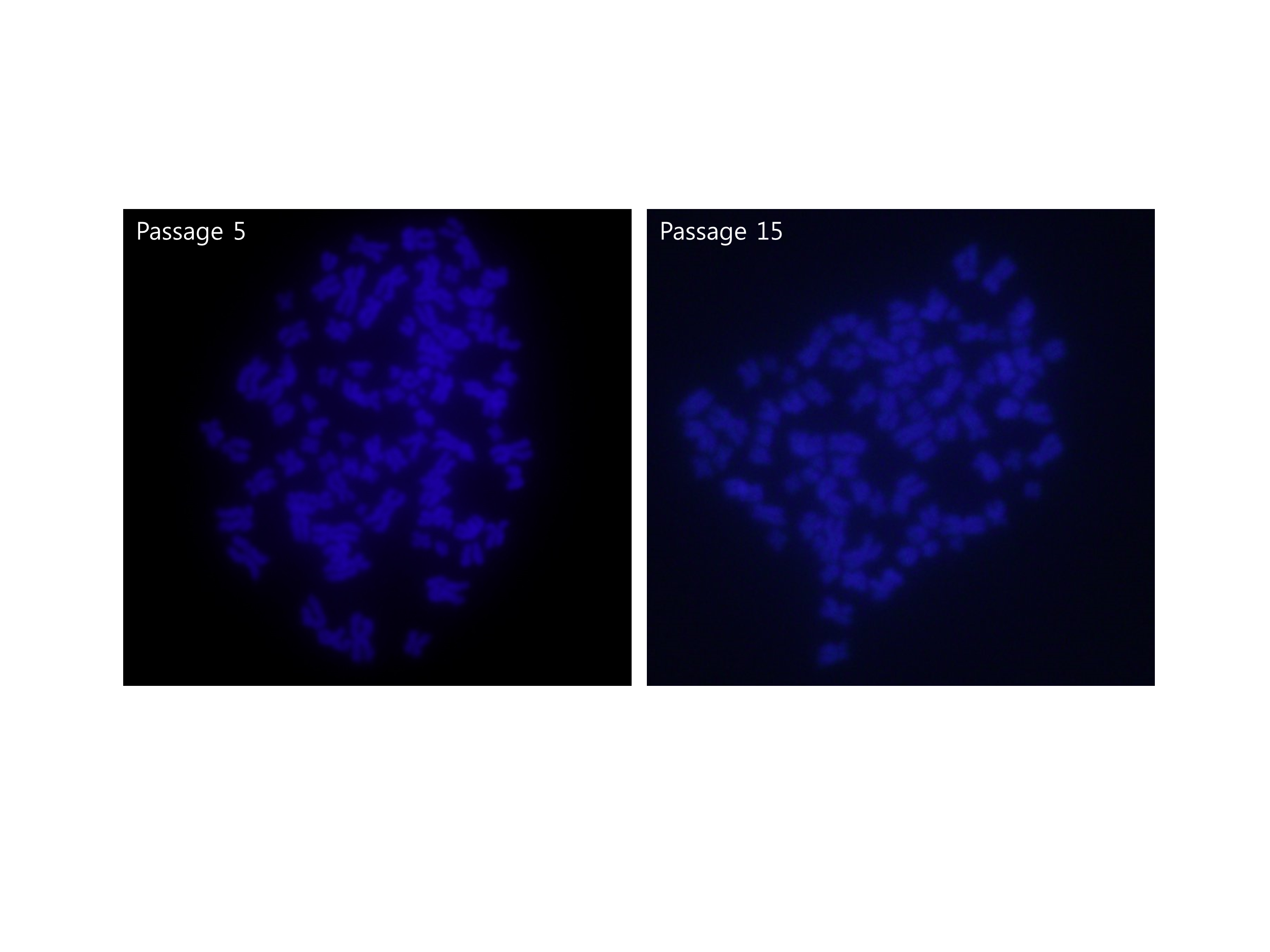

Supplement: Supplementary file 1 [file CAM4-8-3339-s001.tif]
