# Supplementary material for: Establishment of pancreatic cancer cell lines with endoscopic ultrasound‐guided biopsy via conditionally reprogrammed cell culture
Source: Cancer Med. 2019 May 1;8(7):3339–48. doi: 10.1002/cam4.2210 (PMC6601705; doi:10.1002/cam4.2210)
Supplement: Supplementary file 2 [file CAM4-8-3339-s002.pdf]

Table S1. Relationship between procurement of the histologic core and the number of needle passes.

Total number of passes		n (%)
2 passes	Total patients	16
	Sample with a score ≥ 3	14 (87.5%)
3 passes	Total patients	14
	Sample with a score ≥ 3	14 (100%)
Overall	Total patients	30
	Sample with a score ≥ 3	28 (93.3%)

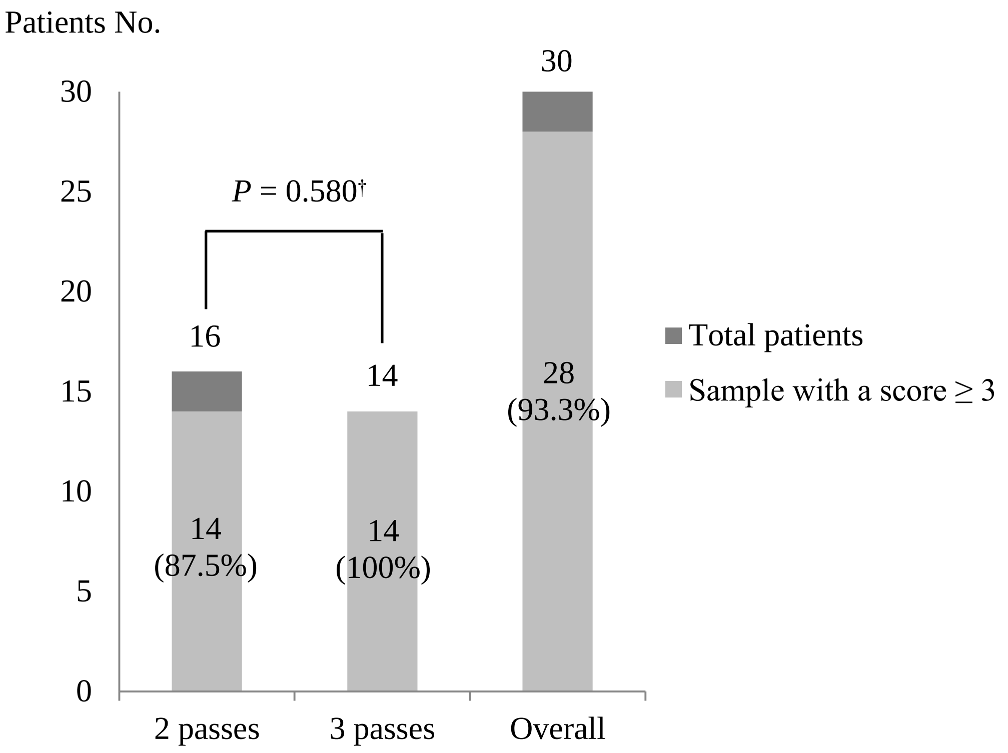

†Mann-Whitney's U test
